# Supervising physicians’ perceptions on physician work-hour regulations in Japan: a nationwide cross-sectional study

**DOI:** 10.1186/s12909-025-08023-8

**Published:** 2025-10-24

**Authors:** Hirohisa Fujikawa, Hidetaka Tamune, Yuji Nishizaki, Kiyoshi Shikino, Taro Shimizu, Yu Yamamoto, Miwa Sekine, Kazuya Nagasaki, Hiroyuki Kobayashi, Yasuharu Tokuda

**Affiliations:** 1 Center for General Medicine Education, School of Medicine, Keio University, 35 Shinanomachi, Shinjuku-ku, Tokyo, 160-8582 Japan; 2 Department of Medical Education Studies, International Research Center for Medical Education, Graduate School of Medicine, The University of Tokyo, Bunkyo-ku, Tokyo, Japan; 3 Present Address: Department of General Medicine, Juntendo University Faculty of Medicine, 2-1-1 Hongo, Bunkyo-ku, Tokyo, 113-8421 Japan; 4 Department of Psychiatry and Behavioral Science, Juntendo University Graduate School of Medicine, Bunkyo-ku, Tokyo, Japan; 5 Division of Medical Education, Juntendo University School of Medicine, Bunkyo-ku, Tokyo, Japan; 6 Department of Community-Oriented Medical Education, Graduate School of Medicine, Chiba University, Chiba, Chiba Japan; 7 Department of Diagnostic and Generalist Medicine, Dokkyo Medical University Hospital, Mibu-machi, Shimotsuga-gun, Tochigi, Japan; 8 Division of General Medicine, Center for Community Medicine, Jichi Medical University, Shimotsuke, Tochigi Japan; 9 Department of Internal Medicine, Mito Kyodo General Hospital, University of Tsukuba, Mito, Ibaraki Japan; 10 Muribushi Okinawa for Teaching Hospitals, Urasoe, Okinawa Japan; 11 Tokyo Foundation for Policy Research, Minato-ku, Tokyo, Japan

**Keywords:** Supervising physician, Supervisor, Working hour restriction, Working hour regulation

## Abstract

**Purpose:**

Japan introduced physician work-hour regulations in April 2024. Perceptions of such regulations appear to be influenced by history and culture, and thus vary among stakeholders and countries. Here, we aimed to investigate supervising physicians’ perceptions of physician work-hour regulations in Japan.

**Methods:**

A nationwide cross-sectional study was conducted from March to April, 2024. We distributed an online anonymous self-administered questionnaire, which included closed questions about expected influence of physician work-hour regulations on various factors and an open-ended question regarding expectations or concerns about the regulations. The data were analyzed using descriptive statistics for the closed questions and inductive content analysis for the open-ended question. To explore whether various factors (sex, postgraduate years, specialty, hospital location, hospital type, and hospital size) were associated with the perceptions of the pariticpants on the implementation of physician work-hour regulations (overall, patient care, resident physician well-being, resident physician education, or supervising physician well-being), we also performed multivariable linear regression analysis.

**Results:**

We included 144 participants in the analysis. Many of the participants raised concerns about the negative impact of the regulations, particularly on the training of medical residents. About one-fifth of the respondents described their thoughts about the gap between the real medical field and the system. Some participants used the Japanese term *jikokensan*, which literally means self-improvement but is used to refer to study or research conducted by a physician with the aim of acquiring knowledge or enhancing skills apart from their primary duties, in the context of physician work-hour regulations. In multivariable analyses, several organizational factors showed statistically significant associations with supervisors’ perceptions of physician work-hour regulations. Nevertheless, effect sizes were small, there was no clear dose–dependent relationships, and findings were inconsistent across the five outcome domains; thus, no strong or consistent predictors were identified.

**Conclusions:**

These results highlight the need to more fully explore supervisor perspectives, which could lead to more discourse in policy-making and an improved system of physician work-hour regulations.

**Supplementary Information:**

The online version contains supplementary material available at 10.1186/s12909-025-08023-8.

## Background

Prolonged working hours have been a part of the routine of physicians across the globe. Although the traditional work week around the world is 40 h [1], physicians typically work more than that. For example, a recent U.S. study demonstrated that the mean number of hours worked per week reported by physicians and workers in other occupations was 50.8 h and 40.7 h, respectively (*p* < 0.001) [2]. However, long working hours can have harmful consequences. Overworking has been identified as one of the most stressful aspects of practicing medicine [3]. Long working hours are associated with burnout among physicians and other healthcare professionals [4, 5]. Chronic work of 52 h or more per week is associated with increased risk of cardiovascular disease, cancer, and poor self-reported general health [6]. In addition, long work hours among physicians can endanger patients; Barger et al.’s nationwide prospective cohort study indicated that longer working hours were associated with greater risk of medical error and injury to patients [7]. Weaver et al. also showed that prolonged weekly working hours were associated with greater risk of adverse safety outcomes independent of cohort [8]. Thus, the long work hours of physicians are a serious issue that may lead to undesirable consequences, and need to be addressed.

One effective strategy for reducing the number of physician working hours is to limit their working hours. This is the most widely used strategy around the world [9]. Beginning in the 1990s, substantial public attention given to the harsh working conditions of medical residents led to the implementation of resident work-hour regulations in Western countries [9]. The European Working Time Directive (EWTD) became law in 1998, limiting working hours to protect the health and safety of workers in all occupations in the European Union [10]. In the U.S., the Accreditation Council for Graduate Medical Education (ACGME) first published working hour standards for medical residents in 2003, and revised the rules in 2011 and 2017 [11]. Implementation of physician working hour limitations has spread to Asia, with Korea and Taiwan beginning to regulate working hours of physicians in the 2010s [12, 13]. Further, in Japan, following considerable social concern about the prolonged working hours of physicians [14, 15], physician work-hour regulations – also referred to as physician work-style reform – were introduced beginning in April, 2024 [16–18].

Researchers have explored the perspectives of medical residents and those of supervising physicians in countries where physician working hour limitations have been implemented. Maisonneuve et al. surveyed on all 1999–2000 medical school graduates in the U.K. in 2021, and showed that approximately 10% agreed that EWTD was beneficial for senior physicians, and that approximately one-third agreed that EWTD was beneficial for junior physicians [19]. Antiel et al. surveyed surgical interns and showed that interns were more optimistic towards the implications of the rules implemented by ACGME than program directors [20]. Han and Chung examined the perspectives of resident physicians and faculty members on the implementation of work-hour regulations in Korea in 2017, and found that faculty members were more concerned about decreased patient safety due to the discontinuity of patient care and inadequate resident education, whereas residents were more concerned about the difficulty in finishing unchanged workloads within the limited duty hours [21]. These examples highlight the importance of verifying the viewpoints of key stakeholders for policy-making. Nevertheless, study findings vary from country to country, underscoring the importance of researching while respecting the history and culture of the country concerned.

Historically, Japanese culture and ethical principles have been shaped by Confucianism, Buddhism, Daoism, and Shintoism [22, 23]. These traditions emphasize harmony, respect for hierarchy, and communal success. They have profoundly influenced Japanese work ethics, in which loyalty, diligence, *ganbaru* (persevering to the best of one’s ability), and *gaman* (quietly enduring hardship with dignity) are widely regarded as moral virtues [24]. These prevailing work ethics have fostered a labor culture that prioritizes collective commitment over individual needs, frequently legitimizing extended working hours as a visible expression of dedication [24]. This appears to contrast with many Western approaches, which tend to emphasize clear boundaries between work and personal life to promote work-life balance [25]. Within the medical profession, these Japanese cultural norms and work ethics have been deeply embedded, shaping physicians’ attitudes toward work.

Patient care in Japan thus has been supported by the dedicated work of physicians, especially young physicians in their 20s and 30s [15, 26]. 2024 regulations on working hours are intended to cover all physicians, and with this historical background, it is assumed that working hours will be largely regulated, especially for young physicians. As in other countries, there is concern that reduced working hours of young physicians will directly translate into more work for others to do, and it appears easy to assume that supervising physicians will make up the deficit [27]. In addition, supervising physicians should teach young physicians effectively during limited working hours. Therefore, in Japan, it is expected that the work style of supervising physicians will change significantly before and after the implementation of physician work-hour regulations. To our knowledge, however, no studies in Japan have examined the perceptions of supervising physicians. Further, it is also important to explore factors associated with these supervisor perceptions, which have also remained under-researched.

Thus, our research questions (RQs) were as follows: (1) How do supervising physicians think about the expected impact of physician work-hour regulations? (2) What are the factors associated with the recognition of the supervisors? To our knowledge, this study is the first to elucidate supervising physicians’ views on physician work-hour regulations in Japan. We expect that it will contribute to discourse for policy-making and improvement of the system of the regulations.

## Methods

### Design, setting, and participants

To facilitate understanding of the context of this study, a description of the Japanese medical education system is herein provided. Subsequent to the completion of medical school and acquisiton of a medical license, medical trainees progress to a two-year early residency program [28]. During the early residency period, trainees are required to rotate through multiple clinical departments [28]. At the end of the academic year, many trainees take part in the General Medicine In-Training Examination (GM-ITE). The GM-ITE is a nationwide in-training examination that was developed by the Japan Institute for Advancement of Medical Education Program (JAMEP; a nonprofit organization) in 2011 to assess the level of clinical knowledge of medical residents. Although the GM-ITE is voluntary [29], it is the only in-training examination taken by medical residents across Japan, with over half of them choosing to take it [30–32]. Upon completion of the early residency program, they transition to a specialty training program that typically extends over a period of three to five years [28].

We conducted a nationwide cross-sectional study in Japan from March 1 to April 30, 2024. We distributed an online self-administered questionnaire to all early residency training program administrators whose programs participated in the GM-ITE. The JAMEP has an extensive network covering the whole of Japan. There are approximately 1000 hospitals in Japan that offers early residency training programs [28]. A total of 696 program administrators from these hospitals were invited to participate in the study. Of these administrators, 634 were from community hospitals, 37 were from university hospitals, and 25 were from university branch hospitals. All of the administrators were supervising physicians.

Prior to participating in the study, the participants read the research document, which informed them of the anonymous and voluntary nature of the study. There were no exclusion criteria and no refusal to consent.

### Survey

The questionnaire (supplementary file 1) used in this study was developed for the following three steps. First, with reference to previous studies [21, 33, 34], the first author drafted a preliminary version of the questionnaire. Second, the research team members, all of whom had rich experiences as supervising physicians in early residency programs, checked the draft and iteratively discussed, resulting in its subsequent revision. Third, a comprehensive review of the questionnaire was conducted, and it was determined that there were no items that were difficult to comprehend or respond to.

We asked the participants about the impact they expected of physician work-hour regulations on the following 13 items, which were rated on a 3-point Likert scale (1 = negative (worse or decreased), 2 = neutral (unchanged), or 3 = positive (better or increased)) and were classified into 4 categories: patient care (3 items; patient safety, patient care quality, and patient care continuity), resident physician well-being (3 items; quality of life of resident physicians, fatigue of resident physicians, and amount of rest of resident physicians), resident education (3 items; number of patients seen by resident physicians, patient care ownership of resident physicians, and resident education quality), and supervising physician well-being (4 items; quality of life of supervising physicians, fatigue of supervising physicians, amount of rest of supervising physicians, and task load of supervising physicians). In our dataset, Cronbach’s alpha values were 0.77 for the 13 items, 0.87 for the 3 items of the patient care category, 0.81 for the 3 items of the resident physician well-being category, 0.77 for the 3 items of the resident education category, and 0.89 for the 4 items of the supervising physician well-being category. These values were above 0.70 and thus indicated good internal consistency reliability [35], which justified the four-category classification employed. We also asked the participants what they expected the various impacts of physician working hour regulations to be in general. This item was also answered with three options: 1 = negative, 2 = neutral, or 3 = positive. Then, we also asked the participants to answer an optional free-text question “Please feel free to describe any expectations or concerns regarding physician work-hour regulations.”

### Data analysis

We conducted a mixed methods approach to analyze our data quantitatively and qualitatively. To examine RQ1, we first reported descriptive statistics for responses to the closed questions. The mean and standard deviation were calculated for each item in accordance with Sullivan and Artino’s recommendation for the interpretation of Likert-type scale data [36].

For analysis of the open-ended question, we analyzed qualitatively each response using inductive content analysis [37, 38]. The authors adopted Haggarty et al.’s definition of content analysis, as follows: “a research method which allows the qualitative data collected in research to be analyzed systematically and reliably so that generalizations can be made from them in relation to the categories of interest to the researcher” [39]. In performing content analysis, we referred to the method of Hawking et al. [40] and proceeded in five steps. First, the first and second author read the responses several times for better understanding. Second, they independently generated initial codes inductively. Third, they reviewed the codes and discussed them iteratively until all discrepancies were resolved. Fourth, they were reviewed by all authors and revised accordingly. All authors confirmed them. Codes were classified into themes, category, and sub-category based on similarities and differences. Fifth, we calculated the percentage of each theme, category, and sub-category to show its relative importance in the overall picture. We also showed illustrative examples.

To elucidate RQ2, we performed multivariable linear regression analysis to explore the associations of various factors with the recognition of the supervisors. We treated the Likert-type scale responses to each close-ended question as continuous variables in our model. This approach was supported by a substantial body of literature demonstrating that such treatment does not affect statistical analysis [36, 41–43]. The mean of the 13 items and each of the four categories (i.e., patient care, resident physician well-being, resident physician education, and supervising physician well-being) were thus calculated and each was used as an outcome variable in separate regression models. Explanatory variables included several individual (sex, postgraduate years, and specialty) or program (hospital location, hospital type, and hospital size) factors. The assumption of residual normality was tested and considered as satisfied. We chose complete case analysis because of the small percentages of participants with missing data. We considered a two-tailed p-value below 0.05 statistically significant in this study. All quantitative analysis was performed using SPSS version 29.0 (IBM Corp), while content analysis was conducted using Microsoft Excel version 16.100.1.

### Ethical considerations

The study was approved by the ethics committee of JAMEP (23–29). All participants provided informed consent before participating in the study. This study was conducted according to the ethical standards and principles of the Declaration of Helsinki.

## Results

A total of 151 (21.7%) responses were returned from 696 eligible participants. Of these, 7 participants with missing data were excluded, and the remaining 144 participants were included in the analysis (i.e., complete case analysis). Table 1 shows a profile of the participants. 128 (88.9%) were male and 130 (90.3%) belonged to a community hospital.

**Table 1:** Profile of the study participants (*n* = 144)

Characteristic	n (%)
Sex	
Female	16 (11.1)
Male	128 (88.9)
Unanswered	0 (0)
Postgraduate years	
10–19	13 (9.0)
20–29	48 (33.3)
30–39	71 (49.3)
≥40	12 (8.3)
Specialty	
Internal medicine and pediatrics	94 (65.3)
Surgery medicine	42 (29.2)
Others	8 (5.6)
Hospital location	
Rural	95 (66.0)
Urban	49 (34.0)
Hospital type	
Community hospital	130 (90.3)
University hospital	8 (5.6)
University branch hospital	6 (4.2)
Hospital size	
< 300 beds	21 (14.6)
300–399 beds	34 (23.6)
400–499 beds	41 (28.5)
≥500 beds	48 (33.3)

### RQ1. How do supervising physicians think about the expected impact of physician work-hour regulations?

Table 2 shows descriptive statistics of the closed-questions. Approximately one-third of the respondents anticipated that patient care would be impaired (e.g., continuity of patient care, 51 (35.4%)). Approximately 40–50% of the respondents expressed concerns about a possible deterioration in the quality of resident education (e.g., number of patient seen by residents, 69 (47.9%)). The proportion of respondents who indicated that supervising physician well-being would result in a negative outcome exceeded the proportion of respondents who indicated that supervising physician well-being would result in a positive outcome (e.g., task load of supervising physicians, 48 (33.3%) vs. 5 (3.5%)). Thus, we noted a trend that participants were concerned about the negative effects of work-hour regulations, although with the contrasting response that the regulations would improve resident well-being.

**Table 2:** Participant predictions of the effects of physician work-hour regulation (*n* = 144)

Question	Survey response, n (%)			Mean (standard deviation)
	1 = Worse	2 = Unchanged	3 = Better	
How will the following be affected by physician work-hour regulations?				
Patient care				
Patient safety	32 (22.2)	106 (73.6)	6 (4.2)	1.82 (0.48)
Quality of patient care	45 (31.3)	94 (65.3)	5 (3.5)	1.72 (0.52)
Continuity of patient care	51 (35.4)	91 (63.2)	2 (1.4)	1.66 (0.50)
Resident physician well-being				
Quality of life of resident physicians	10 (6.9)	69 (47.9)	65 (45.1)	2.38 (0.61)
Fatigue of resident physicians	6 (4.2)	58 (40.3)	80 (55.6)	2.51 (0.58)
Amount of rest of resident physicians	5 (3.5)	66 (45.8)	73 (50.7)	2.47 (0.57)
Resident education				
Number of patients seen by residents	69 (47.9)	74 (51.4)	1 (0.7)	1.53 (0.52)
Patient care ownership of resident physicians	64 (44.4)	79 (54.9)	1 (0.7)	1.56 (0.51)
Quality of education for resident physicians	61 (42.4)	82 (56.9)	1 (0.7)	1.58 (0.51)
Supervising physician well-being				
Quality of life of supervising physicians	34 (23.6)	80 (55.6)	30 (20.8)	1.97 (0.67)
Fatigue of supervising physicians	38 (26.4)	79 (54.9)	27 (18.8)	1.92 (0.67)
Amount of rest of supervising physicians	34 (23.6)	87 (60.4)	23 (16.0)	1.92 (0.63)
Task load of supervising physicians	48 (33.3)	91 (63.2)	5 (3.5)	1.70 (0.53)
	Survey response, n (%)			Mean (standard deviation)
	Negative	Neutral	Positive	
Overall, what do you expect to be the impact of the regulations on physician work hours?	56 (38.9)	73 (50.7)	15 (10.4)	1.72 (0.64)

Seventy-one participants responded to the open-ended question. Table 3 shows the content analysis of the responses to this question. We also show illustrative quotes in supplementary table (supplementary file 2). While only approximately one-third expressed expectations, approximately 90% were categorized as concerns. Among concerns, “Limitations of education and growth” was the most common, with 26 (36.6%) comments. 13 (18.3%) were comments about “Gap between the real medical field and the system.” Several participants expressed their opinions by using the Japanese term *jikokensan* (self-improvement; explained in detail below).

**Table 3:** The results of content analysis of responses to the open-ended question regarding expectations or concerns about physician work-hour regulations (*n* = 71)

Theme^a^, *n* (%)	Category^a^, *n* (%)	Sub-category^a^, *n* (%)
Concerns, 62 (87.3)	Limitations of education and growth, 26 (36.6)	Limitations of educational opportunities, 11 (15.5) Physician growth anxiety, 8 (11.3) Deterioration of patient care ownership of young physicians, 7 (9.9)
	System and its impact, 11 (15.5)	Misuse of the system, 3 (4.2) Questions of effectiveness of the system, 3 (4.2) Sustainability of the medical care system, 2 (2.8) Institutional compliance with appearances only, 1 (1.4) Young physicians imitate senior physicians who have little awareness of the system’s implementation, 1 (1.4) Increase in labor costs, 1 (1.4)
	Insufficient human resources/unbalanced staffing, 10 (14.1)	Overload on supervising physicians, 6 (8.5) Physician maldistribution, 2 (2.8) Physician shortage, 1 (1.4) Shortage of people in charge of healthcare measures, 1 (1.4)
	Working environment and motivation, 8 (11.3)	The line of working hours, 2 (2.8) Avoidance of overtime work more than necessary, 1 (1.4) Even common-sense working may be considered overwork, 1 (1.4) Increase in unreported overtime work, 1 (1.4) Reduced flexibility in working styles, 1 (1.4) Generation gap, 1 (1.4) Decreased motivation of physicians, 1 (1.4)
	Patient care quality and impact of focus on efficacy, 7 (9.9)	Deterioration of patient care quality, 6 (8.5) Important things lost by focusing on efficiency, 1 (1.4)
Expectations, 22 (31.0)	Physician workstyle and their health, 10 (14.1)	Physician well-being improvement, 7 (9.9) Reduction in physician working hours, 1 (1.4) Improvement of junior resident mental health, 1 (1.4) Junior resident growth through appropriate workload, 1 (1.4)
	Operations and efficiency, 6 (8.5)	Improvement of work efficiency, 3 (4.2) Organizing working content, 2 (2.8) Efficient medical education, 1 (1.4)
	Healthcare team and division of roles, 4 (5.6)	Task shifting, 3 (4.2) Promotion of team-based care, 1 (1.4)
	Social understanding and equality, 2 (2.8)	Improved understanding of physician workstyle among non-healthcare professionals, 1 (1.4) Addressing gender inequality, 1 (1.4)
Others, 25 (35.2)	Gap between the real medical field and the system, 13 (18.3)	Gap between the real medical field and the system, 13 (18.3)
	Proposal for compensation, 4 (5.6)	Proposal for compensation commensurate with work, 3 (4.2) Proposal to change payroll system, 1 (1.4)
	Education and career challenges, 2 (2.8)	The challenges for senior and junior residents are different, 1 (1.4) Utilization of *jikokensan*^b^ is key, 1 (1.4)
	Human resources and society, 2 (2.8)	Proposal to increase the number of physicians, 1 (1.4) Need for society to tolerate the possibility of deterioration in patient care quality, 1 (1.4)
	Change and adaptation, 2 (2.8)	Instability during transition period, 1 (1.4) No major changes, 1 (1.4)
	Others, 2 (2.8)	Lack of understanding, 1 (1.4) Unclear, 1 (1.4)

^a^Total counts are greater than 71 because some responses were coded multiple times across different themes, categories, or sub-categories

^b^*Jikokensan* means self-improvement

###  RQ2. What are the factors associated with the recognition of the supervisors?

We performed multivariable linear regression analysis to explore factors associated with the perception of the participants (Table 4). Supervisors in university branch hospitals had more negative perceptions on physician work-hour regulations than those in community hospitals (adjusted mean difference: −0.28, 95% confidence interval: −0.52 to −0.04). Supervisors working in hospitals with 300–399 beds (adjusted mean difference: −0.23, 95% confidence interval: −0.39 to −0.07) and 500 beds or more (adjusted mean difference: −0.17, 95% confidence interval: −0.32 to −0.01) exhibited more negative perceptions, compared to those working in hospitals with less than 300 beds. However, no clear dose-dependent relationship (e.g., increasing number of hospital beds were associated more negative perceptions on the regulations among supervisors) was observed. Additionally, the results varied across the five outcome variables (i.e., overall, patient care, resident physician well-being, resident physician education, and supervising physician well-being).

**Table 4:** Multivariable linear regression analysis exploring factors associated with supervising physicians’ perceptions of physician work-hour regulations (*n* = 144)

Outcomes	Adjusted mean difference	95% CI
Total (Mean 1.90, SD 0.29)		
Sex		
Male	Ref.	Ref.
Female	0.05	-0.11 to 0.20
PGY		
10–19	Ref.	Ref.
20–29	-0.04	-0.22 to 0.15
30–39	-0.02	-0.19 to 0.17
≥40	0.04	-0.20 to 0.27
Specialty		
Internal medicine and pediatrics	Ref.	Ref.
Surgery medicine	0.03	-0.08 to 0.14
Others	0.19	-0.02 to 0.40
Hospital location		
Rural	Ref.	Ref.
Urban	-0.05	-0.15 to 0.06
Hospital type		
Community hospital	Ref.	Ref.
University hospital	-0.00	-0.24 to 0.23
University branch hospital	-0.28*	-0.52 to -0.04
Hospital size		
< 300 beds	Ref.	Ref.
300–399 beds	-0.23**	-0.39 to -0.07
400–499 beds	-0.11	-0.26 to 0.05
≥500 beds	-0.17*	-0.32 to -0.01
Patient care (Mean 1.73, SD 0.45)		
Sex		
Male	Ref.	Ref.
Female	0.06	-0.17 to 0.30
PGY		
10–19	Ref.	Ref.
20–29	-0.18	-0.45 to 0.10
30–39	-0.05	-0.32 to 0.22
≥40	-0.03	-0.39 to 0.32
Specialty		
Internal medicine and pediatrics	Ref.	Ref.
Surgery medicine	0.02	-0.15 to 0.19
Others	0.32*	0.01 to 0.63
Hospital location		
Rural	Ref.	Ref.
Urban	0.06	-0.09 to 0.21
Hospital type		
Community hospital	Ref.	Ref.
University hospital	0.20	-0.15 to 0.55
University branch hospital	-0.21	-0.57 to 0.15
Hospital size		
< 300 beds	Ref.	Ref.
300–399 beds	-0.46**	-0.70 to -0.22
400–499 beds	-0.15	-0.38 to 0.08
≥500 beds	-0.35**	-0.59 to -0.12
Resident physician well-being (Mean 2.46, SD 0.50)		
Sex		
Male	Ref.	Ref.
Female	0.07	-0.20 to 0.35
PGY		
10–19	Ref.	Ref.
20–29	-0.23	-0.55 to 0.10
30–39	-0.36*	-0.69 to -0.04
≥40	-0.31	-0.72 to 0.11
Specialty		
Internal medicine and pediatrics	Ref.	Ref.
Surgery medicine	0.04	-0.15 to 0.24
Others	-0.02	-0.39 to 0.36
Hospital location		
Rural	Ref.	Ref.
Urban	-0.13	-0.31 to 0.04
Hospital type		
Community hospital	Ref.	Ref.
University hospital	-0.12	-0.53 to 0.30
University branch hospital	-0.34	-0.76 to 0.09
Hospital size		
< 300 beds	Ref.	Ref.
300–399 beds	-0.00	-0.29 to 0.28
400–499 beds	0.01	-0.26 to 0.29
≥500 beds	0.02	-0.25 to 0.31
Resident physician education (Mean 1.56, SD 0.42)		
Sex		
Male	Ref.	Ref.
Female	-0.00	-0.23 to 0.23
PGY		
10–19	Ref.	Ref.
20–29	-0.03	-0.29 to 0.24
30–39	0.04	-0.23 to 0.30
≥40	0.25	-0.10 to 0.59
Specialty		
Internal medicine and pediatrics	Ref.	Ref.
Surgery medicine	0.01	-0.15 to 0.18
Others	0.32*	0.01 to 0.63
Hospital location		
Rural	Ref.	Ref.
Urban	-0.06	-0.21 to 0.09
Hospital type		
Community hospital	Ref.	Ref.
University hospital	0.02	-0.32 to 0.37
University branch hospital	-0.32	-0.67 to 0.04
Hospital size		
< 300 beds	Ref.	Ref.
300–399 beds	-0.21	-0.44 to 0.03
400–499 beds	-0.08	-0.31 to 0.15
≥500 beds	-0.15	-0.39 to 0.08
Supervising physician well-being (Mean 1.88, SD 0.54)		
Sex		
Male	Ref.	Ref.
Female	0.05	-0.25 to 0.35
PGY		
10–19	Ref.	Ref.
20–29	0.20	-0.15 to 0.56
30–39	0.24	-0.12 to 0.59
≥40	0.20	-0.26 to 0.66
Specialty		
Internal medicine and pediatrics	Ref.	Ref.
Surgery medicine	0.03	-0.18 to 0.25
Others	0.16	-0.25 to 0.57
Hospital location		
Rural	Ref.	Ref.
Urban	-0.05	-0.24 to 0.15
Hospital type		
Community hospital	Ref.	Ref.
University hospital	-0.09	-0.54 to 0.37
University branch hospital	-0.27	-0.73 to 0.20
Hospital size		
< 300 beds	Ref.	Ref.
300–399 beds	-0.23	-0.54 to 0.08
400–499 beds	-0.19	-0.50 to 0.11
≥500 beds	-0.19	-0.49 to 0.12

*Abbreviation*: *CI* Confidence interval, *PGY* Postgraduate years, *Ref.* Reference category, *SD* Standard deviation

**p < 0.01

*p < 0.05

## Discussion

We performed a cross-sectional study of supervising physicians’ perceptions of physician work-hour regulations in Japan. Many of the participants were concerned about the regulations’ negative impact, especially on resident physician education. Approximately one-fifth of the respondents described their thought about the gap between the real medical field and the system. The Japanese term *jikokensan* was used by some participants. Exploratory analysis showed no consistent or obvious trend. The findings of the present study will contribute to discourse for policy-making and improvement of the system of physician work-hour regulation.

### RQ1. How do supervising physicians think about the expected impact of physician work-hour regulations?

The finding that a large number of supervisors expressed concern is consistent with the results of research conducted outside of Japan [21, 44, 45]. According to a U.S. study conducted one year after implementation of the ACGME 2011 duty hour reform, 60% of the internal medicine program directors thought that the reform had led to a deterioration in resident education [44]. Wyman et al. performed a qualitative study focused on consultant surgeons that showed their concerns about lack of educational experience among current surgical trainees [45]. A Korean study also showed that in the era of work hour regulations, faculty members were more concerned about residents’ professional development than were the residents themselves [21]. Thus, the finding that supervising physicians are worried about the impact of physician work hour regulation on resident education appears to be an international phenomenon.

The results of qualitative analysis offered profound insights into the findings provided by the quantitative analysis in the present study. It is noteworthy that a considerable number of supervising physicians described their thoughts about the gap between the real medical field and the system. This would have two implications. First, the authors are concerned that the discrepancy between actual medical practice and the system of work-hour regulations may lead to a violation of established working hours. This is reflected by the emergence of subcategories, including “Institutional compliance with appearances only” and “Misuse of the system.” In fact, outside Japan, there is substantial evidence to show that resident physicians often violate work-hour regulations [46–49]. The second is the importance of listening to the key stakeholder who will be most affected by the implementation of physician work-hour regulations: the supervising physicians. Although the 2024 work hour regulations apply to all physicians in Japan, it is expected that the reduction in work hours for younger physicians will directly result in more work for other physicians, particularly supervisors. This is also reflected by the emergence of the “Overload on supervising physicians” subcategory in our content analysis. The views of this important stakeholder – the supervising physician – will need to be more fully investigated in future policy-making.

This study also notes the influence of the country-specific context that shapes attitudes to work in Japan, as illustrated by the term *jikokensan*. This term is defined as study or research conducted by a practicing physician to acquire knowledge or improve skills apart from their primary duties, such as medical treatment [17]. However, as shown in the exemplar quote of the subcategory “The line of working hours,” it appears to be difficult to distinguish *jikokensan* and work, and physicians throughout Japan are struggling with this difficult issue. As *jikokensan* sometimes obscures the boundary between regulated duty hours and activities considered personal professional development, it represents a culturally specific challenge that may complicate compliance. Thus, any consideration of physician working hour regulations must take into account the country-specific context. In particular, the effect of *jikokensan* should be explicitly considered in future policy design in Japan.

### RQ2. What are the factors associated with the recognition of the supervisors?

Our exploratory analysis indicated that several organizational factors had statistically significant associations with supervisor perceptions of physician work-hour regulations. Larger hospitals may encounter greater administrative challenges in complying with the regulations, which could partially explain the more negative perceptions among their supervising physicians. However, it is noteworthy that the effect sizes were small, and the overall pattern was not consistent. Taken together, we concluded that the analysis did not identify strong or consistent predictors. These weak or heterogeneous associations may be attributable to several factors, including limited statistical power due to sample size limitations, measurement issues of the instrument used, and a genuine lack of robust associations between the explored individual/environmental factors and perceptions. Future studies should include more samples to achieve sufficient statistical power. In addition, future questionnaires should investigate supervisors’ individual and hospital-related factors in greater detail. These factors may include amount of time spent on direct patient care and amount of time spent on direct resident physician education. These factors may be significantly associated with perceptions of physician work-hour regulations.

### Limitations

The present study was subject to some potential limitations. First, although the questionnaire had been developed with reference to previous studies [21, 33, 34] and had been used for laypeople and medical students [50, 51], it was not formally validated among supervising physicians. In addition, the 13 survey items were grouped into four categories, and linear regressions analysis was employed for ordinal data (Likert-type responses). Future research is needed to validate the questionnaire and our statistical approach, which would confirm the significance of our study. Second, the response rate was relatively low. The response rate is comparable to those of other published surveys of faculty members on this topic [21]. Additionally, the ratio of hospital types where supervising physicians were affiliated (community hospital: university hospital: university branch hospital) was nearly identical between the entire group to whom questionnaires were distributed (634:37:25) and the group of respondents (130:8:6), suggesting that the distribution of hospital types among respondents was representative of the target population. However, supervisors with a less pronounced interest in medical education and occupational health of physicians were less likely to respond to the questionnaire. If so, our study may have been subject to bias. Caution is thus required when interpreting the results of the study. Third, there may be social desirability bias; if present, however, this was likely greatly minimized by making the questionnaire anonymous.

### Implications

Despite the presence of these potential limitations, the study offers essential implications. First, implementation strategies should address supervisors’ major concerns—such as potential deterioration in patient care, resident education, and workload—while also clarifying how activities regarded as *jikokensan* should be positioned in relation to regulated working hours. Future policies should explicitly evaluate and communicate how educational quality, professional growth, and supervisors’ well-being can be supported within regulated hours, as this will be essential to reducing resistance and fostering trust in the system. Second, views on physician work-hour regulations greatly vary across stakeholders. A recent study on laypeople in Japan showed that they tend to emphasize the potential enhancement of physician well-being as expectations for the regulations [50]. Another study of Japanese medical students found their mixed views, including both expectations for enhanced physician well-being and concerns for increase in *sabisu zangyo* (unpaid work) and limited opportunities for the growth as physicians [51]. The reconciliation of these disparate viewpoints is imperative for the formulation of educationally sound and socially legitimate policies regarding work-hour regulations.

## Conclusions

This nationwide cross-sectional study investigated the perceptions of supervising physicians on the implementation of physician work-hour regulations. The results show that many of the participants were worried about the implementation’s negative influence, particularly on education for medical residents. About one-fifth of the participants described their thoughts about a gap between the real medical field and the system. The findings of this study underscore the need to examine the perspectives of supervising physicians more fully, which could lead to further improvements to the physician work hour regulation system.

## Supplementary Information

Supplementary Material 1.

Supplementary Material 2.

## Data Availability

Only upon reasonable request, the corresponding author can provide the data sets generated and analyzed in the study.

## Abbreviations

ACGME: Accreditation Council for Graduate Medical Education
EWTD: European Working Time Directive
GM-ITE: General Medicine In-Training Examination
JAMEP: Japan Institute for Advancement of Medical Education Program
