# Supplementary material for: Supervising physicians’ perceptions on physician work-hour regulations in Japan: a nationwide cross-sectional study
Source: BMC Med Educ. 2025 Oct 24;25:1489. doi: 10.1186/s12909-025-08023-8 (PMC12551151; doi:10.1186/s12909-025-08023-8)
Supplement: Supplementary file 1 — Supplementary Material 1. [file 12909_2025_8023_MOESM1_ESM.pdf]

Supplementary file 1: Questionnaire examining supervising physicians' perception of physician work-hour regulations in Japan

How will the following be affected by physician working hour restrictions? Please select one of the options.

- Patient safety: worse, unchanged, or better
- Quality of patient care: worse, unchanged, or better
- Continuity of patient care: worse, unchanged, or better
- Quality of life of resident physicians: worse, unchanged, or better
- Fatigue of resident physicians: worse, unchanged, or better
- Amount of rest of resident physicians: decreased, unchanged, or increased
- Number of patients seen by residents: decreased, unchanged, or increased
- Patient care ownership of resident physicians: worse, unchanged, or better
- Quality of education for resident physicians: worse, unchanged, or better
- Quality of life of supervising physicians: worse, unchanged, or better
- Fatigue of supervising physicians: worse, unchanged, or better
- Amount of rest of supervising physicians: decreased, unchanged, or increased
- Task load of supervising physicians: decreased, unchanged, or increased

Overall, what do you expect to be the impact of physician work-hour regulations?: bad, neutral, or good

Please feel free to describe any expectations or concerns regarding physician work-hour regulations (optional free-text question).
