# Supplementary material for: Supervising physicians’ perceptions on physician work-hour regulations in Japan: a nationwide cross-sectional study
Source: BMC Med Educ. 2025 Oct 24;25:1489. doi: 10.1186/s12909-025-08023-8 (PMC12551151; doi:10.1186/s12909-025-08023-8)
Supplement: Supplementary file 2 — Supplementary Material 2. [file 12909_2025_8023_MOESM2_ESM.pdf]

Supplementary file 2

Supplementary table. Exemplar quotes of responses to open-ended question regarding expectations or concerns about physician work-hour regulation (n = 71)

Theme	Category	Sub-category and illustrative quotes
Concerns	Limitations of education and growth	Limitations of educational opportunities<br>*"(Working hour restrictions will) put work restrictions on the period of greatest growth (as a physician)."*<br>Physician growth anxiety<br>*"I believe that the quality, knowledge, and skills of physicians will decline."*<br>Deterioration of patient care ownership of young physicians<br>*"I am concerned that the sense of responsibility towards patients will decline among young physicians."*
	System and its impact	Misuse of the system<br>*"(I am concerned that) the 'work-style reform' will only curb the apparent number of working hours, and (that) the number of undeclared working hours will balloon."*<br>Questions of effectiveness of the system<br>*"(Regulating physician working hours) is only going to end up confusing medicine."*<br>Sustainability of the medical care system<br>*"I am concerned about whether medical care will be viable when there is only a new generation of physicians, because until now medical care has been supported by the volunteer spirit of physicians."*<br>Institutional compliance with appearances only<br>*"However, I remain concerned about whether the system (of regulating physician working hours) is really compliant and workable…if it is too prescriptive and a large number of physicians do not comply, it could lead to a potential problem."*<br>Young physicians imitate senior physicians who have little awareness of the system's implementation<br>*"I am concerned that senior physicians are unaware of the need to comply with the work-style reform, and that junior residents are copying them and ignoring their working hours."*<br>Increase in labor costs<br>*"(I am concerned about) an increase in labor costs."*
	Insufficient human resources/unbalanced staffing	Overload on supervising physicians<br>*"If the work of young physicians is reduced, the bill will be paid only by senior physicians."*<br>Physician maldistribution<br>*"Physicians will become more and more unevenly distributed by region, department, and hospital, with physicians gathering in places with good quality of life and those with poor quality of life becoming worse and worse, resulting in polarization."*<br>Physician shortage

		"We need more physicians."<br>Shortage of physicians in charge of healthcare measures<br>*"There are no physicians that are in charge of healthcare measures."*
	Working environment and motivation	The line of working hours<br>*"How to distinguish and draw the line between jikokensan*[a] *and work is a difficult question."*<br>Avoidance of overtime work more than necessary<br>*"While it is welcome that resident work hours are managed and appropriate to reduce burnout and depression, all residency programs feel that overtime work should not be allowed."*<br>Even common-sense working may be considered overwork<br>*"I am concerned that even common-sense working may be considered overwork."*<br>Increase in unreported overtime work<br>*"(I am concerned that) burakku*[b] *hospital will become more burakku."*<br>Reduced flexibility in working style<br>*"I will not be able to do things that have been flexible. I will no longer be able to help others (e.g., married women)."*<br>Generation gap<br>*"There is likely to be some friction for a few years between people who operate by the old standards and the younger generation."*<br>Decreased motivation of physicians<br>*"I am concerned that the motivation of physicians (especially supervising physicians) will be decreased."*
	Patient care quality and impact of focus on efficacy	Deterioration of patient care quality<br>*"While the quality of life of physicians will improve, the quality of patient care will inevitably decline."*<br>Important things lost by focusing on efficiency<br>*"I am afraid that if we focus on efficiency, we will lose sight of what is important."*
Expectations	Physician workstyle and their health	Physician well-being improvement<br>*"I believe that physician overwork will improve."*<br>Reduction in physician working hours<br>*"(I expect that) physician working hours will be reduced."*<br>Improvement in junior resident mental health<br>*"While it is welcome that resident work hours are managed and appropriate to reduce burnout and depression…"*<br>Junior resident growth through appropriate workload<br>*"We expect (resident physician's) growth through training within appropriate overtime hours as an important period for learning the basics of being a physician and growing as a professional."*
	Operations and efficiency	Improvement of work efficiency<br>*"I expect that work efficiency will be improved by the work-style reform…"*<br>Organizing working content

		"I believe that we should prioritize efforts to reduce unnecessary work and maximize time spent on truly necessary medical care."<br>Efficient medical education<br>*"I am very much aware of the need to make (medical) education more efficient."*
	Healthcare team and division of roles	Task shifting<br>*"(Regulation of physician working hours) is a strong impetus for more task shifting."*<br>Promotion of team-based care<br>*"(I expect that) team-based care will be promoted."*
	Social understanding and equality	Improved understanding of physician workstyle among non-healthcare professionals<br>*"I hope that the public will understand more and that it will be easier for physicians to take time off."*<br>Addressing gender inequality<br>*"(Regulation of physician working hours) is definitely necessary to address the gender inequality among physicians…"*
Others	Gap between the real medical field and the system	Gap between the real medical field and the system<br>*"It will be impossible to just reduce working hours when the workload is increasing."*
	Proposal for compensation	Proposal for compensation commensurate with work<br>*"I believe that there must be a system of fair payment for overtime work."*<br>Proposal to change payroll system<br>*"I think that it is difficult to promote (the regulation of physician working hours) if the salary system is 'the longer you work, the more you earn.' I think the salary should be based on the quantity and quality of work performed."*
	Education and career challenges	The challenges for senior and junior residents are different<br>*"Hard to answer when asked about senior and junior residents in the same sentence."*<br>Utilization of *jikokensan*[a] is key<br>*"Utilization of jikokensan is key…it would be good to have a common tool for junior residents to study away from work."*
	Human resources and society	Proposal to increase the number of physicians<br>*"The current number of physicians is unlikely to be able to work in the way the government has indicated. We need 1.5 to 2 times the number of...physicians who will be forced to reduce their work."*<br>Need for society to tolerate the possibility of deterioration in patient care quality<br>*"…the quality of patient care will inevitably decline. Society needs to tolerate it."*
	Change and adaptation	Instability during transition period<br>*"How to get through the 5 years it will take to stabilize this system is critical."*

		No major changes<br>*"Since the reforms (in our hospital) four years ago until now, there have been no significant changes in the practice, fatigue, or lives of the resident and supervising physicians."*
	Others	Lack of understanding<br>*"I cannot understand."*

[a] *Jikokensan* means self-improvement

[b] *Burakku kigyo* is the Japanese term for a company that overworks its employees for low (or even no) wages.
